# Spontaneous Heterotopic Pregnancy: A Case Report

**DOI:** 10.31729/jnma.8374

**Published:** 2023-12-31

**Authors:** Sujata Maharjan, Rumina Malla, Babita Chaudhary, Piyush Shrestha, Lakpa Dolma Lama

**Affiliations:** 1 Department of Obstetrics and Gynaecology, Kathmandu Medical College and Teaching Hospital, Sinamangal, Kathmandu, Nepal; 2 Department of Obstetrics and Gynaecology, Nepalese Army Institute of Health Sciences, Sanobharyang, Kathmandu, Nepal; 3 Department of Radiology, Kathmandu Medical College and Teaching Hospital, Sinamangal, Kathmandu, Nepal

**Keywords:** *case reports*, *ectopic pregnancy*, *laparoscopy*, *ultrasound*

## Abstract

Spontaneous heterotopic pregnancy is a rare clinical condition which is a potentially dangerous condition where at least two pregnancies are present simultaneously at different implantation sites and only one is located in the intrauterine cavity. It is a life-threatening condition with an incidence estimated as 1 in 30,000 natural conceptions. Being rare it's challenging to diagnose such conditions due to complex clinical and laboratory findings. In view of the survival of maternal as well as intrauterine pregnancy, a high index of suspicion leading to timely diagnosis and appropriate intervention is needed. We are reporting a case of a 28-year-old female with heterotopic pregnancy at 8 weeks of gestation following natural conception diagnosed by ultrasound and managed successfully by laparoscopic salpingectomy. Intrauterine pregnancy was continued normally till term with no complications. Hence, with timely diagnosis and early intervention, maternal and fetal survival is possible.

## INTRODUCTION

Heterotopic pregnancy (HP) is a rare complication of pregnancy, in which both intrauterine and extrauterine gestation occur simultaneously.^1^ The incidence of spontaneous heterotopic pregnancy in the natural conception is estimated as 1 in 30000.^2^ With widespread assisted reproductive techniques (ART), the incidence is increased to about 0.09-1.00%.^3,4^ It is challenging to diagnose HP even with the advancement and availability of high-resolution ultrasound imaging and Doppler techniques due to complex clinical and laboratory findings.^5^ We report a case of spontaneous HP of 8 weeks gestation which was managed with laparoscopic salpingectomy while intrauterine pregnancy was continued successfully until she delivered a healthy baby.

## CASE REPORT

A 28-year-old, female primigravida, presented to the Outpatient Department of Kathmandu Medical College, Sinamangal with complaints of amenorrhea for 2 months, per vaginal spotting for 3 weeks, and pain in the abdomen occasionally. She had confirmed her pregnancy 2 weeks before presentation by urine pregnancy test at home. The bleeding was spotting type just staining her undergarments and was associated with pain lower abdomen. She had no history of recent sexual intercourse, trauma, or instrumentation. Her last menstrual period was 8 weeks back with a history of regular menstrual cycle. Family history of twin pregnancy present. There is no significant past medical and surgical history. There is no history of ovulation induction and in vitro fertilization as well.

On abdominal examination, it was soft and there was tenderness over the right iliac fossa but no rebound tenderness, and no organs were palpable. Speculum examination revealed a healthy cervix with blood-stained discharge. No polyp or cervical mass suggestive of a local lesion causing vaginal bleeding was noted. During vaginal examination, uterine size could not be assessed due to guarding but cervical motion tenderness was present.

**Figure 1:**
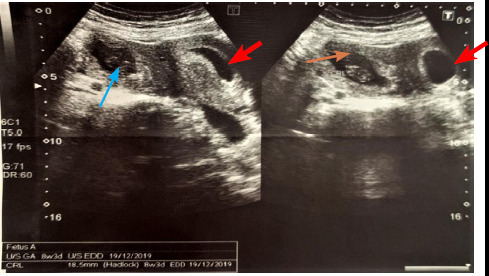
Transabdominal ultrasonography showing intrauterine gestation sac (red arrow) and right adnexal ectopic pregnancy with the single yolk sac (blue arrow) and single embryonic pole of 18.5 mm without cardiac activity (orange arrow).

Following examination, she was urgently advised for ultrasonography. Transabdominal ultrasonography was performed on Toshiba Aplio 400 with a convex probe which revealed a gestational sac with a single yolk sac and single embryonic pole in the right adnexal region measuring 18.5 mm by crown rump length (CRL), corresponding to gestational age of 8 weeks and 3 days without cardiac activity (Figure 1).

**Figure 2:**
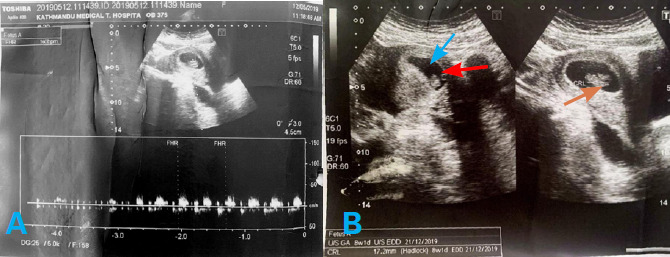
Transabdominal ultrasonography with pulsed wave Doppler interrogation. A) intrauterine gestation sac and embryonic pole with a fetal heart rate of 160 bpm, B) intrauterine gestation sac (blue arrow) with single yolk sac (red arrow) and single embryonic pole of 17.2mm (orange arrow).

Single intrauterine gestational sac with single yolk sac and single live embryo of CRL 17.2 mm corresponding to gestation age of 8 weeks and 1 day with normal cardiac activity (Figure 2).

The right ovary was separately visualised. The left ovary and adnexal region were unremarkable. Mild anechoic free fluid was noted in Pouch of Douglas. The conclusion of heterotopic pregnancy with single live intrauterine pregnancy and right-sided ectopic pregnancy without cardiac activity was reported. She was planned for a diagnostic laparoscopy. Preoperative investigations were sent which were within normal range. With high risk was informed with written consent, and she was transferred to the operating theatre. General anaesthesia was given. Laparoscopy was done. During laparoscopy, multiple flimsy adhesions were noted between the omentum and anterior abdominal wall so adhesiolysis was done. The right tube and ovary were adherent with mesosalpinx. Right ampullary ectopic mass extending up to the infundibulum on the verge of tubal abortion was noted. Left tube and ovary adherent posteriorly with the uterus. The uterus was around 8 weeks in size. There was hemoperitoneum around 300 ml. Right salpingectomy was done and the specimen was sent for histopathological examination.

The histopathology report showed a salpingectomy specimen measuring 5.5×2 cm. A portion of the fallopian tube dilated measuring 3.5 cm in length with multiple fragments of tissues. Microscopic examination revealed chorionic villi and clusters of trophoblastic cells in the lumen and wall in the section from the fallopian tube. Large areas of haemorrhage and fibrinous materials were noted. Her postoperative events were uneventful. She was given prophylactic antibiotics and was started on micronized progesterone soft gelatin capsules 200 mg per vaginally kept twice daily for 4 weeks. She was discharged on postoperative day and was followed up after 2 weeks.

**Figure 3:**
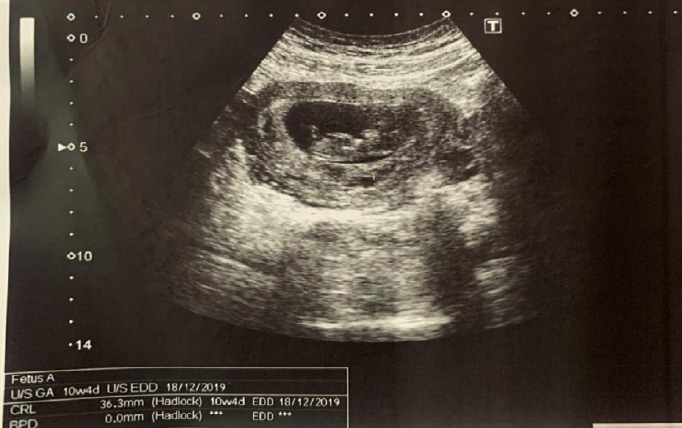
Transabdominal ultrasonography showed a single live intrauterine pregnancy of 10 weeks and 4 days with CRL measuring 36.3 mm.

After 2 weeks of the postoperative period, her stitches were removed. The port site was intact and healthy. Ultrasonography was advised which revealed single live intrauterine pregnancy of 10 weeks and 4 days gestation (Figure 3).

She was followed up as per the World Health Organization (WHO) antenatal care protocol including maternal and fetal well-being. Her antenatal period was uneventful. Pregnancy was continued till 38 weeks of gestation. She presented with prelabour rupture of the membrane and was induced. She delivered a single live female baby weighing 3500 gm. Her postpartum period was uneventful and discharged in second postoperative day.

## DISCUSSION

Heterotopic pregnancy is a rare complication of pregnancy with incidence estimated as 1 in 30000 natural conceptions.^2^ Early diagnosis and management of ectopic pregnancy reduce the high probability of tubal rupture leading to a life-threatening situation. Early diagnosis of heterotopic pregnancy is difficult but the clinician needs to be vigilant. In most scenarios, HP can be asymptomatic but may also present with vaginal bleeding, abdominal pain, or features of shock due to hypovolemia. These clinical features are evident only in case of tubal rupture.^6^ HP can also present with hematometra and lower quadrant pain.^7^ HP was diagnosed in our case before rupture and fortunately before landing into maternal shock.

HP or ectopic pregnancy can simulate intrauterine pregnancy with hemorrhagic corpus luteum both clinically and by ultrasound. Bicornuate uterus with gestation in both horns mimics HP. Similarly, other surgical conditions of the acute abdomen can also simulate heterotopic gestation clinically further causing difficulty in clinical diagnosis.^8^ In our context, the patient didn't present with severe abdominal pain while vaginal spotting was one of the triggering factors leading to ectopic pregnancy as the diagnosis.

A high-resolution transvaginal ultrasonography with colour Doppler will be helpful as the trophoblastic tissue in the case of heterotopic pregnancy shows increased flow with significantly reduced resistance index which is an important aid in the diagnosis of the heterotopic pregnancy.^9^ Similarly we were able to diagnose HP timely with the help of ultrasonography. After diagnosis, HP is managed surgically either by laparoscopy or laparotomy. An ectopic component is usually removed surgically, while the intrauterine component is expected to develop normally. The least invasive procedure is selected to preserve intrauterine pregnancy as much as possible.^10^ Since, the case presented with stable vitals, laparoscopy was planned as a minimally invasive procedure to continue intrauterine pregnancy safe and sound.

Hence, heterotopic pregnancy being a rare condition, is most likely to be missed in natural conception. For pregnant women presenting with abdominal pain and adnexal abnormality, heterotopic pregnancy should be considered as one of the differential diagnoses. Thorough diagnosis through investigation using the available most common resource ultrasound should be done. It is better to exclude this rare diagnosis and allow timely management before presentation with rupture and acute abdominal syndrome which can progress to maternal shock leading to maternal mortality.
